# Cancer Survival by Stage at Diagnosis in Kuwait: A Population-Based Study

**DOI:** 10.1155/2019/8463195

**Published:** 2019-09-09

**Authors:** E. Alawadhi, A. Al-Awadi, A. Elbasmi, M. P. Coleman, C. Allemani

**Affiliations:** ^1^Cancer Survival Group, London School of Hygiene & Tropical Medicine, London, UK; ^2^Kuwait Cancer Control Center, Ministry of Kuwait, Kuwait City, Kuwait

## Abstract

**Objective:**

To examine the distribution of stage at diagnosis for 12 cancers in Kuwait, to estimate stage-specific net survival at 1 and 5 years after diagnosis, and to assess differences in stage-specific survival between Kuwait and the United States.

**Material and Methods:**

Data were obtained from the Kuwait Cancer Registry, for Kuwaiti patients diagnosed during 2000–2013, with follow-up to 31 December 2015. The distribution of Surveillance Epidemiology and End Results (SEER) Summary Stage for 12 malignancies was examined. We estimated net survival by stage up to 5 years after diagnosis, controlling for background mortality with life tables of all-cause mortality in the general population by single year of age, sex, and calendar period. Survival estimates were age-standardised using the International Cancer Survival Standard (ICSS) weights.

**Results:**

Only 14.2% of patients were diagnosed at a localised stage and 38.9% at the regional stage. The proportion of patients with known stage was 88.9% during 2000–2004 but fell to 59.4% during 2010–2013. During 2005–2009, 1- and 5-year survival for colon, rectal, breast, cervical, and prostate cancer was about 90% or higher for patients diagnosed at the localised stage. During 2004–2009, the proportion of patients diagnosed at a localised stage was lower in Kuwait than in the US for colon, breast, and lung cancer. Age-standardised 5-year net survival for all stages combined was lower in Kuwait than the US for colon, lung, and breast cancer, but stage-specific survival was similar.

**Conclusion:**

Since stage-specific survival is similar in Kuwait and the US, late stage at diagnosis is likely to be a major contributing factor to the overall lower survival in Kuwait than in the US. Increasing public awareness of cancer risk factors and symptoms and investment in early detection will be vital to reduce the proportion of patients diagnosed at a late stage and to improve survival.

## 1. Introduction

Stage at diagnosis, the anatomic extent of a disease, is a major determinant of patients' outcomes. [1] It is crucial in predicting patients' prognosis and to inform treatment decisions, as well as to assess the effect of public health interventions such as screening programmes and educational or awareness campaigns, which aim to improve early-stage diagnosis. Stage information is also valuable to help plan the provision of cancer-related resources and services, to monitor compliance to treatment guidelines, and to offer more detailed analyses of cancer outcomes [2].

Evaluation of the distribution of stage at diagnosis helps to assess the intensity of diagnostic activity in a given country or region. Examination of population-based survival trends by stage at diagnosis helps to determine the effectiveness of the health system in offering optimal, stage-specific treatment to all patients. In Kuwait, net survival was lower than that in other high-income countries [3]. Differences in the distribution of stage at diagnosis are likely to be a key determinant of these discrepancies. The distribution of stage at diagnosis for each cancer can also reflect the level of symptom awareness as well as the thoroughness of the staging procedures within a region or country [4].

Population-based cancer survival by stage has never been assessed in Kuwait. In order to provide a better understanding of cancer survival in the country, our study aims to assess the distribution of stage at diagnosis in Kuwait for 12 cancers for which data are available and to estimate stage-specific net survival at 1 and 5 years since diagnosis. Differences in stage-specific survival between Kuwait and the United States will also be assessed.

## 2. Materials and Methods

We obtained data from the Kuwait Cancer Registry for all adult Kuwaiti patients (aged 15–99 years) diagnosed during 2000–2013 with one of 18 malignancies [3, 5]. Data on stage were available for 12 cancers: oesophagus, stomach, colon, rectum, liver, pancreas, lung, melanoma, breast (women), cervix, ovary, and prostate. All tumours were defined by anatomical site (topography) and coded to the International Classification of Diseases for Oncology (third edition, ICD-O-3) [6] and its first revision [7].

Data were assessed for quality and completeness according to the protocol and standardised quality-control procedures from the CONCORD programme for global surveillance of cancer survival [8]. Records considered ineligible for survival analyses were excluded. Full details of exclusions and data quality indicators have been published [3].

Follow-up data were available until 31 December 2015. Information on follow-up was obtained using a new method [9] combining active and passive follow-up procedures, which has been shown to be highly effective in ascertaining each patient's vital status. Complete dates of death of deceased cancer patients were obtained from the Central Records Department of Births and Deaths, at Kuwait's Ministry of Health. When the vital status could not be ascertained, the patients were considered lost to follow-up and were censored from survival analyses.

We present the distribution of stage at diagnosis for the 12 malignancies based on the Surveillance Epidemiology and End Results (SEER) Summary Stage 2000, [10] which categorises the extent of the disease as localised, regional (with lymph node involvement, or direct extension, or both), or distant metastasis.

Patients were grouped into 3 consecutive calendar periods (2000–2004, 2005–2009, and 2010–2013). We estimated stage-specific net survival only for cancers with at least 10 patients in each stage category and calendar period. Due to low numbers for most cancers, we present unstandardised 1- and 5-year stage-specific survival estimates for all ages combined.

Standardisation is crucial when comparing populations that differ with respect to age. Due to Kuwait's relatively small population and the rarer nature of some cancers, however, age-standardisation of survival was only possible for three cancers: colon, lung, and breast. To make comparisons between Kuwait and the US, stage-specific survival estimates were obtained from the CONCORD-2 supplementary studies on US data for colon, [11] lung [12], and breast cancer [13]. To be able to compare results in Kuwait with those in the US, survival was estimated for the calendar period 2004–2009. For these analyses, we present age-standardised stage-specific 5-year net survival, where possible.

Net survival is the probability for cancer patients to survive their cancer up to a given time following diagnosis (e.g., 1 or 5 years), after correcting for competing causes of death (background mortality). To control for background mortality, we used life tables of all-cause mortality in the general population. We used life tables by single year of age (“complete” life tables), sex, calendar year of death, and nationality (Kuwaiti; non-Kuwaiti) [8].

We used the Pohar–Perme estimator [14] to estimate net survival, implemented with the programme stns [15] in Stata version 14 [16]. This estimator accounts for the fact that the hazard of death due to causes other than cancer (competing causes) is higher among older patients.

For patients diagnosed during 2000–2003 and 2004–2009, the *cohort* approach was used to estimate survival. The cohort approach is considered the gold standard [17] and can be used only when all patients in the cohort have had the opportunity to be followed up for the full duration of the follow-up required, in this case, five years. For patients diagnosed during 2010–2013, the *complete* approach was used because five years of follow-up data were not available for all patients by December 2015. This approach enables survival estimates to be produced for recently diagnosed patients [18].

Net survival estimates were age-standardised where possible, using the International Cancer Survival Standard (ICSS) weights, [19] in which age at diagnosis is categorised into 5 groups: 15–44, 45–54, 55–64, 65–74, and 75–99 years. The 95% confidence intervals (CI) for all unstandardised and age-standardised estimates were derived assuming a normal distribution, truncated to the range 0–100. Confidence intervals were constructed using standard errors calculated using the Greenwood method [20]. When no deaths or censorings occurred within 5 years, or if all patients died (survival probability 1 or 0), a binomial approximation was obtained for the upper and lower bounds of the CI.

## 3. Results

Colon (46.6%), rectal (39.7%), breast (49.4%), and cervical cancer (36.2%) were most commonly diagnosed at regional stage, while liver (29.9%), pancreas (48.3%), and lung (41.2%) were mostly diagnosed at distant stage (Table 1). For oesophagus (∼23%), stomach (∼32%), melanoma (∼22%), and ovary (∼32%), the proportion of stage at diagnosis was similar for both regional and distant stages. The proportion of men diagnosed with prostate cancer at localised stage (25.7%) was similar to the proportion of men diagnosed at distant stage (24.0%).

Overall, stage data were available for 74.1% of patients diagnosed during 2000–2013. This proportion decreased from 88.9% in 2000–2004 to 59.4% in 2010–2013. Over this 14-year period, the highest proportion of unknown stage was for liver (52.1%) and oesophageal cancer (42.2%); the lowest was for colon (19.1%) and breast cancer (21.1%).

Between 2000–2004 and 2005–2009, when the availability of data on stage was reasonably high, a common trend was observed in the stage distribution for most cancers: the proportion of patients diagnosed at localised and regional stage decreased, while that of distant and unknown stage increased. The exceptions were colon and liver cancer, where the proportion of patients diagnosed at localised stage remained similar, and cancers of the breast and colon, where the proportion diagnosed at a localised stage increased slightly (18.7% to 22.4%, and 23.3 to 25.4%, respectively).

### 3.1. Stage-Specific Survival

In general, survival for all cancers was lower for patients diagnosed at more advanced stage (Table 2). For patients diagnosed during 2010–2013, for whom unknown stage at diagnosis was the highest, survival for patients with unknown stage was either similar or higher than the survival of all stages combined, for almost all cancers. This trend was also observed for patients diagnosed with unknown stage during 2000–2004 and 2005–2009.

**Figure 1:**
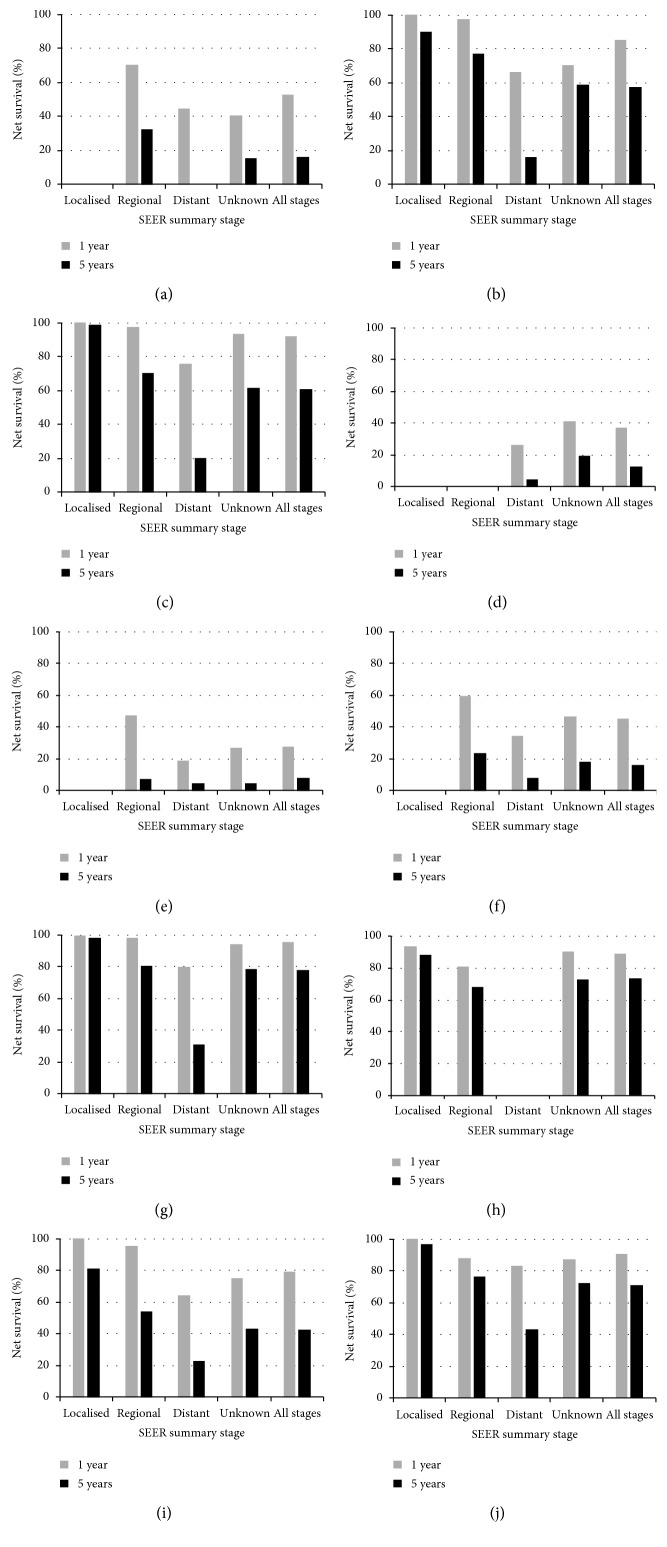
Trends in unstandardised net survival (NS, %) at 1 and 5 years, by SEER Summary Stage at diagnosis, Kuwait 2005–2009. (a) Stomach, (b) colon, (c) rectum, (d) liver, (e) pancreas, (f) lung, (g) breast, (h) cervix, (i) ovary, and (j) prostate.

During 2005–2009, one-year survival for colon, rectal, breast, and ovarian cancer was generally similar for patients diagnosed at localised or regional stage (Figure 1). One- and five-year survival for prostate, rectal, breast, cervical, and prostate cancer was high (almost 90% or higher) and relatively similar, for patients diagnosed at localised stage. Five-year survival for all cancers was relatively low, ranging from 43.5% for prostate to 0% for stomach, for patients diagnosed at distant stage.

During the same period, the greatest difference in five-year survival between regional and distant stage at diagnosis was observed for colon, rectum and breast cancer (>50%), followed by prostate, stomach, and ovarian cancer (about 30%). For some of the more lethal cancers, the difference in one-year survival between regional and distant stage was substantially smaller (around 25%), e.g., for stomach (70.4% vs. 44.7%) and pancreatic cancer (47.1% vs. 18.6%).

### 3.2. Comparisons between Kuwait and the United States

**Figure 2:**
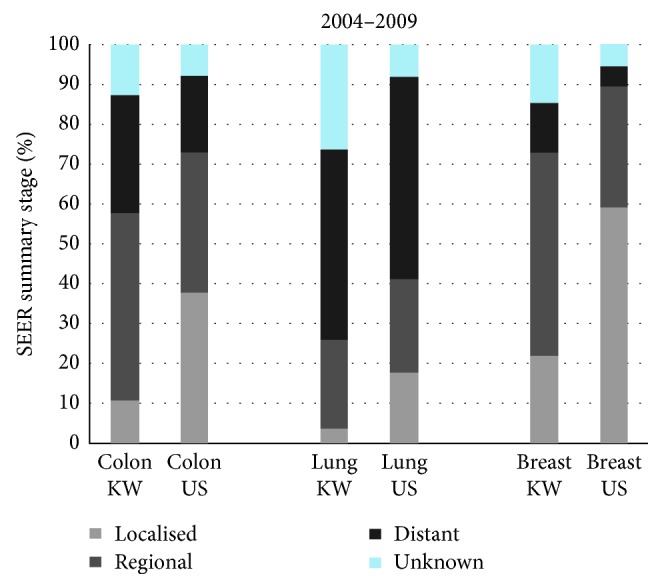
Distribution (%) of SEER Summary Stage for adults in Kuwait (Kuwaiti) and the United States (all races), 2004–2009. SEER SS: Surveillance, Epidemiology, and End Results Summary Stage; KW: Kuwait; US: United States.

During 2004–2009, the proportion of patients diagnosed at localised stage was substantially lower in Kuwait than in the US for colon (10.7% vs. 37.8%) and breast (21.9% vs. 59.1%), while the proportions of regional, distant and unknown stages were higher, with differences ranging from 5% to 21% (Figure 2). For lung cancer, the proportion of localised stage was much lower in Kuwait than in the US (3.5% vs. 17.7%); however, the proportion was similar in the two countries for regional (22.5% vs. 23.4%) and distant stage (47.6% vs. 50.0%).

Age-standardised five-year net survival in Kuwait for all stages combined for colon (50.6%), lung (15.3%), and breast (70.8%) was lower than in the US (64.6%, 19.0%, and 88.6%, respectively) (Table 3).

For colon cancer, stage-specific five-year net survival was similar in Kuwait and the US for both regional disease (73.0% vs. 70.2%) and distant stage (13.7% vs. 13.8%).

For lung cancer, the only age-standardised stage-specific survival estimate available for Kuwait was for distant stage. Survival for patients diagnosed at distant stage in Kuwait was somewhat higher than in the US (8.0% vs. 4.8%). For breast cancer, stage-specific survival was generally similar in Kuwait and the US: slightly lower for localised stage (94.4% vs. 98.3%) and slightly higher for distant stage (28.4% vs. 24.5%). For regional stage, stage-specific survival in Kuwait was lower than in the US (75.7% vs. 82.3%).

## 4. Discussion

This is the first population-based study to date in Kuwait to assess the distribution of stage at diagnosis and stage-specific survival, over a 14-year period, for up to 12 malignancies. Examining the distribution of stage at diagnosis is essential to interpret the variations in survival over time and helps identify cancers for which earlier diagnosis can achieve the greatest benefit. Differences in population-based survival between different populations or regions may also be partly explained by differences in stage of disease at diagnosis. [21, 22] This study produced stage-specific net survival estimates up to 5 years for colon, lung, and breast cancer, taking into account the differences in the age profile of cancer patients and the risk of death from other causes, thus enabling robust comparisons of stage-specific survival over time.

Age-standardisation is essential to compare survival over time, or between different regions, since net survival can vary considerably by age, and the age structure of cancer patients differs between countries and over time. However, due to the small number of patients available for analysis in Kuwait, age-standardisation of the survival estimates for each stage category was not possible for many cancers. Comparisons of stage-specific survival over time in Kuwait were therefore performed using unstandardised estimates.

During 2000–2013, the stage at diagnosis was known for 74% of the patients. The proportion of patients with known stage decreased over the 14-year period, reaching its lowest (59%) during 2010–2013. The mean age and the age distribution were generally similar for patients with known and unknown stage during 2010–2013, except for pancreatic cancer, where the proportion of patients older than 85 years was higher among patients with unknown stage. The survival for patients with known stage, for most cancers, was also generally similar to that for patients with unknown stage. The unavailability of information on stage in this case is therefore less likely due to physicians' staging practices or to patients not being medically fit for staging and treatment. A plausible explanation could be that more patients are receiving their first treatment abroad and therefore are not staged in Kuwait. Receiving treatment abroad is a service provided by the government, covering full treatment costs. With the Ministry of Health's increased budget for overseas treatment in 2009, more patients have been utilising this option [23]. The increased proportion of unknown stage for patients diagnosed during 2010–2013 could thus be due to more patients receiving treatments abroad.

During 2005–2009, 1- and 5-year stage-specific survival for patients diagnosed at localised stage was about 90% or higher for colon, rectal, breast, cervical, and prostate cancer. In Kuwait, these cancers are most commonly diagnosed at regional stage, and the proportion of patients diagnosed at localised stage is low, ranging from 25.4% for prostate to 10.5% for colon cancer. Furthermore, the largest difference in survival was observed between patients diagnosed at regional and distant stages. This difference in five-year net survival was most evident (greater than 50%) for cancers of the colon, rectum, and breast, followed by those of prostate, stomach, and ovary, where this difference was about 30%. This further highlights the cancers for which early diagnosis is important and where greater efforts are essential to ensure that more patients are diagnosed at an earlier stage, particularly for cancers for which early detection tests and procedures are available [24].

For colon, lung, and breast, we compared the survival in Kuwait to that in the US, where the survival is among the highest worldwide [5]. We used the calendar period, cancer definitions, data quality control procedures, and analytical methods used for the CONCORD-2 supplementary analyses of stage-specific survival for the US [25], allowing, therefore, appropriate and robust comparisons.

Stage-specific survival for colon cancer in Kuwait was similar to survival in the US for patients diagnosed at regional and distant stages. Due to low number of patients, it was not possible to estimate stage-specific survival for localised stage in Kuwait; however, the proportion of patients diagnosed at localised stage, which generally entails good prognosis, was substantially higher in the US (37.8%) than in Kuwait (10.7%). This difference in the proportion of patients diagnosed at early stage could partially explain the lower survival for all stages combined observed in Kuwait.

For lung cancer, survival for all stages combined was lower in Kuwait (15.3%) than in the US (19.0%). Comparisons of stage-specific estimates were only possible for distant stage. The proportion of distant stage, however, constitutes the majority of lung cancer patients, which was similar in Kuwait and the US (47.6% and 50.9%, respectively). Stage-specific survival for distant stage was somewhat higher in Kuwait (8.0%) than in the US (4.8%). Therefore, the lower survival for all stages combined in Kuwait is probably attributable to differences in the proportion of patients diagnosed at the localised stage, which was substantially lower in Kuwait (3.5%) than in the US (17.7%). Further investigation is necessary to explain the higher survival for patients diagnosed at distant stage in Kuwait. While the introduction of targeted therapies has improved the treatment of advanced lung cancer, [26] the very high cost of these treatments can limit their adoption and application [27]. In Kuwait, treatment is fully covered by the government, so financial limitations will probably have little effect on the usage of such therapies. In the US, medical insurance coverage can limit patients' access to some of the less cost-effective treatments, particularly for patients with a poor prognosis. Therefore, to understand these differences in stage-specific survival, it would be necessary to assess the differences in the modality and access to treatment between the countries.

For breast cancer, the difference in early stage at diagnosis may explain the lower survival observed for all stages combined between Kuwait (70.8%) and the US (88.6%). The lower proportion of women diagnosed at a localised stage could be due to lack of screening. Unlike the US, screening programmes for breast cancer were not available for women diagnosed during 2004–2009, since screening officially commenced in Kuwait in 2014 [28]. Differences in early stage diagnoses between the two countries could also be due to other factors such as the population's awareness of early symptoms, knowledge of risk factors, and access to timely diagnostic tests.

Survival in Kuwait was also lower than in several other high-income countries [3]. This could also be attributable to differences in diagnostic activity and the tendency towards later diagnosis in Kuwait. Differences in survival can arise due to several other reasons that require further investigation: prevalence of comorbidities; attitudes and behaviours towards treatment; differences in primary care systems; delays in access to treatment; and the efficacy of treatment.

The small population of Kuwait limited our analyses, where the estimation of stage-specific survival by sex was not possible due to the small number of patients available for analysis. The interpretation of stage-specific trends over the 14-year period (2000–2013) was also affected by a high proportion of unknown stage, which was higher for patients diagnosed during 2010–2013 than for patients diagnosed in earlier years.

## 5. Conclusion

Complete information on stage at diagnosis is required in order to assess the effectiveness of cancer control strategies. In Kuwait, the quality of and completeness of stage data can be improved in several ways, the most urgent of which is investing in the Kuwait Cancer Registry. This would include increasing the labour force, enabling the staff to cope with the increasing number of diagnoses and continuously updating the staff's knowledge and skills in order to adapt to changes in staging and coding guidelines. Another way would be to implement more systematic procedures for retrieving patients' medical notes from different hospitals, particularly in the case of those receiving treatment abroad. Finally, investing in an electronic medical record system where all patients' medical data would be stored electronically could improve the timelines substantially and maximise efficiency in accessing patients' data.

This study supplements our previous knowledge on the effect of stage at diagnosis as a major determinant of outcome. Our study also shows that a low proportion of early-stage diagnoses could be a major contributing factor to lower survival in Kuwait than in other high-income countries. Investment in early detection and increasing public awareness of cancer risk factors and symptoms will be vital to reduce the proportion of late-stage diagnoses and, ultimately, improve outcomes.

## Figures and Tables

**Table 1:** Number of patients and distribution of SEER Summary Stage at diagnosis, by cancer and calendar period; Kuwaiti adults (15–99 years).

Cancer site	No. of cases							
	2000–2004		2005–2009		2010–2013		All periods	
	(*n*)	(%)	(*n*)	(%)	(*n*)	(%)	(*n*)	(%)
*Oesophagus*								
Localised	**6**	22.2	**2**	8.3	**3**	7.7	**11**	12.2
Regional	**10**	37.0	**6**	25.0	**4**	10.3	**20**	22.2
Distant	**4**	14.8	**8**	33.3	**9**	23.1	**21**	23.3
Unknown	**7**	25.9	**8**	33.3	**23**	59.0	**38**	42.2
Total	**27**		**24**		**39**		**90**	

*Stomach*								
Localised	**2**	3.4	**2**	2.5	**2**	2.9	**6**	2.9
Regional	**32**	54.2	**23**	29.1	**19**	27.5	**74**	35.8
Distant	**13**	22.0	**34**	43.0	**13**	18.8	**60**	29.0
Unknown	**12**	20.3	**20**	25.3	**35**	50.7	**67**	32.4
Total	**59**		**79**		**69**		**207**	

*Colon*								
Localised	**25**	10.6	**34**	10.5	**25**	7.2	**84**	9.3
Regional	**162**	68.4	**151**	46.5	**110**	31.8	**423**	46.6
Distant	**39**	16.5	**99**	30.5	**90**	26.0	**228**	25.1
Unknown	**11**	4.6	**41**	12.6	**121**	35.0	**173**	19.1
Total	**237**		**325**		**346**		**908**	

*Rectum*								
Localised	**19**	22.4	**18**	13.9	**13**	11.3	**50**	15.2
Regional	**48**	56.5	**53**	40.8	**30**	26.1	**131**	39.7
Distant	**9**	10.6	**31**	23.9	**17**	14.8	**57**	17.3
Unknown	**9**	10.6	**28**	21.5	**55**	47.8	**92**	27.9
Total	**85**		**130**		**115**		**330**	

*Liver*								
Localised	**3**	4.4	**5**	4.7	**4**	4.6	**12**	4.6
Regional	**17**	25.0	**8**	7.6	**10**	11.5	**35**	13.4
Distant	**15**	22.1	**39**	36.8	**24**	27.6	**78**	29.9
Unknown	**33**	48.5	**54**	50.9	**49**	56.3	**136**	52.1
Total	**68**		**106**		**87**		**261**	

*Pancreas*								
Localised	**2**	3.5	**4**	4.4	**1**	1.1	**7**	2.9
Regional	**23**	40.4	**15**	16.5	**15**	16.3	**53**	22.1
Distant	**22**	38.6	**49**	53.9	**45**	48.9	**116**	48.3
Unknown	**10**	17.5	**23**	25.3	**31**	33.7	**64**	26.7
Total	**57**		**91**		**92**		**240**	

*Lung*								
Localised	**9**	5.0	**7**	3.7	**2**	1.1	**18**	3.2
Regional	**78**	43.6	**43**	22.5	**30**	16.0	**151**	27.1
Distant	**66**	36.9	**84**	44.0	**80**	42.6	**230**	41.2
Unknown	**26**	14.5	**57**	29.8	**76**	40.4	**159**	28.5
Total	**179**		**191**		**188**		**558**	

*Melanoma*								
Localised	**1**	20.0	**1**	14.3	**1**	16.7	**3**	16.7
Regional	**2**	40.0	**1**	14.3	**1**	16.7	**4**	22.2
Distant	**1**	20.0	**3**	42.9	**0**	0.0	**4**	22.2
Unknown	**1**	20.0	**2**	28.6	**4**	66.7	**7**	38.9
Total	**5**		**7**		**6**		**18**	

*Breast*								
Localised	**117**	18.7	**213**	22.4	**149**	15.1	**479**	18.7
Regional	**431**	68.9	**461**	48.6	**374**	37.9	**1,266**	49.4
Distant	**47**	7.5	**126**	13.3	**102**	10.3	**275**	10.7
Unknown	**31**	5.0	**149**	15.7	**361**	36.6	**541**	21.1
Total	**626**		**949**		**986**		**2,561**	

*Cervix*								
Localised	**19**	30.7	**15**	25.4	**6**	14.3	**40**	24.5
Regional	**28**	45.2	**20**	33.9	**11**	26.2	**59**	36.2
Distant	**5**	8.1	**3**	5.1	**2**	4.8	**10**	6.1
Unknown	**10**	16.1	**21**	35.6	**23**	54.8	**54**	33.1
Total	**62**		**59**		**42**		**163**	

*Ovary*								
Localised	**10**	16.1	**10**	10.9	**3**	4.5	**23**	10.4
Regional	**30**	48.4	**23**	25.0	**18**	26.9	**71**	32.1
Distant	**16**	25.8	**36**	39.1	**19**	28.4	**71**	32.1
Unknown	**6**	9.7	**23**	25.0	**27**	40.3	**56**	25.3
Total	**62**		**92**		**67**		**221**	

*Prostate*								
Localised	**27**	23.3	**43**	25.4	**61**	27.2	**131**	25.7
Regional	**36**	31.0	**21**	12.4	**15**	6.7	**72**	14.2
Distant	**33**	28.5	**53**	31.4	**36**	16.1	**122**	24.0
Unknown	**20**	17.2	**52**	30.8	**112**	50.0	**184**	36.2
Total	**116**		**169**		**224**		**509**	

*All cancers*								
Localised	**240**	15.2	**354**	15.9	**270**	11.9	**864**	14.2
Regional	**897**	56.7	**825**	37.1	**637**	28.2	**2,359**	38.9
Distant	**270**	17.1	**565**	25.4	**437**	19.3	**1,272**	21.0
Unknown	**176**	11.1	**478**	21.5	**917**	40.6	**1,571**	25.9
Total	**1,583**		**2,222**		**2,261**		**6,066**	

**Table 2:** Unstandardised net survival (NS, %) at 1 and 5 years since diagnosis, by SEER Summary Stage and calendar period; Kuwaiti adults (15–99 years) diagnosed during 2000–2013 and followed up to 31 December 2015.

Cancer site	Calendar period	1-year net survival										5-year net survival									
		Localised		Regional		Distant		Unknown stage		All stages		Localised		Regional		Distant		Unknown stage		All stages	
		NS (%)	95% CI	NS (%)	95% CI	NS (%)	95% CI	NS (%)	95% CI	NS (%)	95% CI	NS (%)	95% CI	NS (%)	95% CI	NS (%)	95% CI	NS (%)	95% CI	NS (%)	95% CI
*Stomach*	2000–2004	—		**53.2**	35.7–70.6	**40.6**	14.6–66.7	**46.6**	18.7–74.6	**50.8**	37.7–63.9	—		**20.7**	6.3–35.1	**11.1**	0.0–29.3	**20.3**	0.0–42.2	**18.9**	8.0–29.9
	2005–2009	—		**70.4**	51.8–88.9	**44.7**	28.2–61.2	**40.3**	19.6–61.1	**52.6**	41.5–63.6	—		**32.1**	13.0–51.1	**0.0**	0.0–0.0	**15.2**	0.6–29.8	**15.9**	7.8–24.0
	2010–2013	—		**59.0**	37.1–80.8	**30.9**	7.5–54.4	**55.2**	38.7–71.7	**51.6**	39.7–63.5	—		**31.9**	9.3–54.4	**8.0**	0.0–19.9	**28.9**	12.2–45.6	**20.6**	8.4–32.9

*Colon*	2000–2004	**100.0**	86.3–100.0	**89.4**	84.3–94.5	**79.7**	66.8–92.5	**90.9**	74.7–100.0	**89.5**	85.2–93.7	**84.6**	66.9–100.0	**69.5**	61.0–77.9	**23.1**	9.2–37.0	**82.4**	52.1–100.0	**64.8**	57.6–72.0
	2005–2009	**100.0**	89.7–100.0	**97.9**	95.1–100.0	**66.2**	56.7–75.8	**70.5**	56.1–85.0	**85.3**	81.3–89.4	**90.0**	76.3–100.0	**77.2**	69.4–84.9	**16.2**	8.8–23.7	**58.8**	41.7–76.0	**57.8**	51.9–63.7
	2010–2013	**100.0**	86.3–100.0	**97.6**	94.1–100.0	**64.2**	54.2–74.3	**87.1**	80.8–93.4	**85.6**	81.8–89.5	**97.6**	80.7–100.0	**83.3**	72.8–93.8	**29.7**	18.5–40.8	**64.6**	51.8–77.4	**63.3**	56.3–70.2

*Rectum*	2000–2004	**89.4**	74.0–100.0	**95.0**	88.1–100.0	—		—		**90.4**	83.5–97.2	**77.4**	54.7–100.0	**53.6**	38.7–68.6	—		—		**60.7**	49.2–72.3
	2005–2009	**100.0**	81.5–100.0	**97.6**	92.4–100.0	**75.6**	60.3–90.9	**93.2**	83.8–100.0	**92.4**	87.3–97.4	**99.0**	88.5–100.0	**70.2**	55.9–84.5	**19.9**	5.2–34.7	**61.5**	41.8–81.2	**60.8**	51.3–70.3
	2010–2013	**100.0**	75.3–100.0	**100.0**	88.4–100.0	**59.8**	36.9–82.7	**94.4**	87.5–100.0	**91.8**	86.3–97.2	**86.6**	65.8–100.0	**76.3**	55.0–97.7	**23.5**	3.0–44.0	**72.7**	45.1–100.0	**67.0**	51.7–82.3

*Liver*	2000–2004	—		**32.2**	10.4–54.0	**27.5**	6.2–48.8	**35.1**	18.8–51.4	**34.3**	22.8–45.8	—		**7.2**	0.0–18.0	**6.9**	0.0–17.4	**9.7**	0.0–20.0	**11.4**	3.5–19.2
	2005–2009	—		—		**26.5**	12.7–40.3	**41.5**	28.3–54.7	**36.8**	27.4–46.1	—		—		**4.4**	0.0–10.5	**19.3**	8.2–30.4	**12.4**	5.8–19.1
	2010–2013	—		**50.5**	21.5–79.5	**29.7**	12.0–47.4	**33.5**	20.2–46.8	**36.5**	26.3–46.8	—		**0.3**	0.0–1.0	**0.0**	0.0–0.0	**11.1**	1.7–20.6	**8.3**	1.8–14.9

*Pancreas*	2000–2004	—		**18.5**	3.3–33.7	**24.7**	7.0–42.3	**40.3**	11.8–68.8	**27.8**	16.0–39.5	—		**9.2**	0.0–20.0	**5.0**	0.0–12.6	**10.1**	0.0–24.9	**11.2**	3.1–19.3
	2005–2009	—		**47.1**	22.8–71.3	**18.6**	8.0–29.3	**26.6**	9.2–44.0	**27.8**	18.6–37.0	—		**6.9**	0.0–17.4	**4.5**	0.0–9.9	**4.4**	0.0–11.1	**8.2**	2.5–13.8
	2010–2013	—		**87.1**	70.4–100.0	**24.8**	12.4–37.2	**46.0**	28.6–63.4	**43.1**	32.9–53.2	—		**12.1**	0.0–28.3	**7.0**	0.1–14.0	**25.0**	6.0–44.1	**15.3**	6.8–23.8

*Lung*	2000–2004	—		**44.9**	33.6–56.2	**29.7**	18.6–40.7	**29.3**	11.7–46.8	**38.0**	30.6–45.3	—		**17.1**	8.1–26.2	**6.8**	0.7–12.8	**8.9**	0.0–19.9	**13.7**	8.2–19.3
	2005–2009	—		**59.8**	44.8–74.8	**34.2**	23.9–44.4	**46.4**	33.2–59.5	**45.0**	37.8–52.2	—		**23.6**	10.0–37.2	**7.6**	1.8–13.5	**17.8**	7.1–28.5	**15.7**	10.1–21.3
	2010–2013	—		**61.5**	43.9–79.2	**39.6**	28.8–50.4	**49.8**	38.5–61.2	**47.9**	40.6–55.1	—		**17.7**	2.3–33.0	**6.7**	0.4–12.9	**19.0**	7.5–30.5	**13.9**	7.6–20.3

*Breast*	2000–2004	**99.0**	96.7–100.0	**96.8**	95.0–98.7	**85.5**	75.2–95.9	**87.6**	75.9–99.2	**96.0**	94.3–97.7	**88.6**	81.4–95.7	**76.7**	72.3–81.2	**44.5**	29.6–59.3	**66.7**	48.8–84.6	**76.1**	72.4–79.9
	2005–2009	**99.3**	97.7–100.0	**98.0**	96.5–99.5	**80.1**	73.0–87.1	**94.3**	90.3–98.4	**95.4**	93.9–96.8	**98.2**	94.4–100.0	**80.8**	76.8–84.9	**30.7**	22.5–39.0	**78.3**	70.3–86.4	**77.7**	74.7–80.7
	2010–2013	**99.7**	98.4–100.0	**97.8**	96.1–99.6	**79.3**	71.3–87.3	**97.3**	95.4–99.2	**96.2**	94.8–97.5	**95.0**	89.2–100.0	**85.4**	80.2–90.7	**38.2**	27.9–48.6	**89.0**	83.7–94.2	**82.6**	79.3–85.9

*Cervix*	2000–2004	**84.6**	68.6–100.0	**89.8**	78.5–100.0	—		**80.2**	56.4–100.0	**84.4**	75.2–93.6	**54.4**	31.9–76.9	**59.3**	40.6–78.0	—		**80.4**	56.6–100.0	**57.8**	45.0–70.6
	2005–2009	**93.6**	81.3–100.0	**80.7**	63.5–97.8	—		**90.7**	78.1–100.0	**88.7**	80.4–97.0	**88.4**	71.5–100.0	**68.3**	47.1–89.4	—		**72.9**	52.5–93.3	**73.8**	61.7–86.0
	2010–2013	—		**91.1**	74.9–100.0	—		**78.6**	61.7–95.4	**86.3**	75.6–96.9	—		**94.7**	77.9–100.0	—		**62.2**	42.1–82.2	**71.8**	57.2–86.5

*Ovary*	2000–2004	**100.0**	69.2–100.0	**77.3**	62.2–92.4	**56.7**	33.7–79.7	**—**		**73.4**	62.2–84.5	**100.0**	69.2–100.0	**36.0**	18.5–53.4	**6.4**	0.0–16.3	**—**		**38.9**	26.3–51.5
	2005–2009	**100.0**	69.2–100.0	**95.8**	87.6–100.0	**64.4**	48.8–80.0	**74.9**	57.2–92.6	**79.0**	70.5–87.4	**81.1**	57.3–100.0	**54.2**	32.6–75.8	**23.1**	9.5–36.7	**43.4**	22.5–64.3	**42.6**	32.0–53.3
	2010–2013	**—**		**84.1**	66.9–100.0	**64.3**	43.0–85.6	**81.9**	67.4–96.3	**78.3**	68.3–88.3	**—**		**61.6**	37.6–85.6	**0.1**	0.0–0.2	**60.6**	41.9–79.3	**40.3**	22.1–58.5

*Prostate*	2000–2004	**100.0**	87.2–100.0	**91.7**	80.8–100.0	**72.4**	56.3–88.6	**88.5**	72.7–100.0	**88.8**	82.0–95.5	**93.3**	69.3–100.0	**88.2**	69.1–100.0	**40.9**	20.6–61.1	**96.9**	72.8–100.0	**78.8**	66.7–90.9
	2005–2009	**100.0**	91.8–100.0	**88.0**	72.8–100.0	**83.6**	72.1–95.0	**87.1**	76.9–97.3	**90.6**	85.3–96.0	**96.6**	80.6–100.0	**76.3**	55.0–97.6	**43.5**	27.1–59.9	**72.5**	55.5–89.4	**71.3**	61.6–80.9
	2010–2013	**100.0**	97.3–100.0	**100.0**	78.2–100.0	**99.5**	91.7–100.0	**94.4**	88.9–99.8	**97.9**	94.7–100.0	**100.0**	100.0–100.0	**56.2**	11.3–100.0	**81.4**	57.2–100.0	**98.3**	84.1–100.0	**98.1**	88.6–100.0

SEER SS: Surveillance, Epidemiology, and End Results Summary Stage; CI: confidence interval.

**Table 3:** Number of patients, SEER Summary Stage distribution (%), and age-standardised 5-year net survival (NS, %), for adults in Kuwait (Kuwaiti) and the United States (all races), 2004–2009.

Cancer	SEER SS	Kuwait			United States		
		No. of patients (%)	NS (%)	95% CI	No. of patients (%)	NS (%)	95% CI
*Colon*	All stages	365	**50.6**	43.4–57.8	534,721	**64.6**	64.4–64.9
	Localised	(10.7)	—	—	(37.8)	**89.7**	89.4–90.0
	Regional	(46.9)	**73.0**	66.8–79.3	(34.9)	**70.2**	69.8–70.6
	Distant	(29.9)	**13.7**	7.7–19.7	(19.3)	**13.8**	13.4–14.1
	Unknown	(12.6)	—	—	(7.9)	**49.4**	48.6–50.2

*Lung*	All stages	227	**15.3**	10.7–20.0	955,184	**19.0**	18.8–19.1
	Localised	(3.5)	—	—	(17.7)	**55.1**	54.7–55.5
	Regional	(22.5)	—	—	(23.4)	**26.4**	26.0–26.7
	Distant	(47.6)	**8.0**	3.8–12.2	(50.9)	**4.8**	4.7–4.9
	Unknown	(26.4)	—	—	(8.0)	**13.8**	13.4–14.3

*Breast*	All stages	1,092	**70.8**	64.0-77.6	926,271	**88.6**	88.4–88.8
	Localised	(21.9)	**94.4**	88.4–100.0	(59.1)	**98.3**	98.1–98.6
	Regional	(51.0)	**75.7**	67.2–84.3	(30.2)	**82.3**	81.9–82.7
	Distant	(12.5)	**28.4**	22.3–34.5	(5.2)	**24.5**	23.7–25.2
	Unknown	(14.7)	**65.7**	53.6–77.9	(5.4)	**72.6**	71.9–73.4

SEER SS: Surveillance, Epidemiology, and End Results Summary Stage; CI: confidence interval.

## Data Availability

The data used to support the findings of this study were supplied by the Kuwait Cancer Registry and so cannot be made freely available. Requests for access to these data should be made to the Ministry of Health at the State of Kuwait.
